# Longitudinal Effects of Group Music Instruction on Literacy Skills in Low-Income Children

**DOI:** 10.1371/journal.pone.0113383

**Published:** 2014-11-19

**Authors:** Jessica Slater, Dana L. Strait, Erika Skoe, Samantha O'Connell, Elaine Thompson, Nina Kraus

**Affiliations:** 1 Auditory Neuroscience Laboratory, Northwestern University, Evanston, Illinois, United States of America; 2 Department of Communication Sciences, Northwestern University, Evanston, Illinois, United States of America; 3 Institute for Neuroscience, Northwestern University, Evanston, Illinois, United States of America; 4 Department of Neurobiology and Physiology, Northwestern University, Evanston, Illinois, United States of America; 5 Department of Otolaryngology, Northwestern University, Evanston, Illinois, United States of America; Birkbeck College, United Kingdom

## Abstract

Children from low-socioeconomic backgrounds tend to fall progressively further behind their higher-income peers over the course of their academic careers. Music training has been associated with enhanced language and learning skills, suggesting that music programs could play a role in helping low-income children to stay on track academically. Using a controlled, longitudinal design, the impact of group music instruction on English reading ability was assessed in 42 low-income Spanish-English bilingual children aged 6–9 years in Los Angeles. After one year, children who received music training retained their age-normed level of reading performance while a matched control group's performance deteriorated, consistent with expected declines in this population. While the extent of change is modest, outcomes nonetheless provide evidence that music programs may have value in helping to counteract the negative effects of low-socioeconomic status on child literacy development.

## Introduction

Music training has been associated with enhanced language and learning skills (see [1], [2] for review), yet a 2011 National Endowment for the Arts survey states that the percentage of 18 year-olds who report receiving music instruction of any kind during childhood (either in school or privately) fell from 53% to 36% between 1982 and 2008 [3]. In other words, *most* children in the United States receive *no* music education. This decline is part of an overall reduction in arts education, particularly in Hispanic and African-American communities, but music has been hardest hit [3]. As policy decisions continue to be made regarding the role of the arts in education, it is relevant to assess the academic benefits of “real world” music programs using a rigorous experimental approach.

Low socioeconomic status can affect the trajectory of reading development [4], [5] through a combination of impoverished home language environment [6] and reduced access to print materials [7]. These factors reduce the amount of reading practice, which in turn inhibits the development of reading fluency and constrains the growth of vocabulary. If strong early reading skills are established, this can bootstrap more rapid development of advanced skills; conversely, if a child starts to fall behind in elementary school, the disadvantage is likely to be compounded by the time the child reaches high school: this reflects the so-called “Matthew effect” [8], with the impact of socioeconomic status on literacy development resulting in an increasing gap in achievement over time between children from low- and high-socioeconomic backgrounds. This effect is further exaggerated in a bilingual population, where the primary language spoken in the home is often not the language in which academic achievement is measured [9].

Large-scale correlational analyses provide evidence that, irrespective of race and socio-economic status, extent of participation in school music programs relates with better performance in many academic areas, including math, reading and SAT scores [10]–[12], as well as higher graduation rates and lower dropout rates [13]. However, there is also evidence that the children who do better academically are more likely to choose and persist with music training [14]. To disambiguate the effects of training from pre-existing differences between those who pursue music training and those who do not, it is necessary to perform random-assignment longitudinal studies in which children are matched at the outset for both educational and motivational factors.

Music and language skills rely upon auditory processing [1], [15]: although reading may not be thought of as a primarily auditory activity, a child must first be able to make sense of incoming auditory input in order to map sounds (phonemes) correctly onto orthographic representations (graphemes) as reading skills develop. Many of the same aspects of sound processing that are deficient in children with language and learning impairments have been found to be strengthened in those who receive music training (for review see [16]), and music-based interventions have demonstrated some success in the remediation of reading problems [17]–[19].

It has been theorized that music training promotes plasticity in speech-processing networks when certain criteria are met, including the precise manipulation of sound as well as emotional engagement, attention and repetitive practice [2], [20]. Enhancements in auditory processing are evident in musicians across the lifespan [21]–[24], even in toddlers who participate in informal music activities at home [25], and advantages for neural processing in musicians have been demonstrated for speech as well as music [1], [22], [24], [26]–[32]. A small number of longitudinal studies have provided further evidence that music training enhancements can transfer to the realm of language processing [28], [33]–[36], though transfer can take time: three recent longitudinal studies demonstrated the emergence of enhanced speech processing after two academic years of music training, but not after a single year [29], [37], [38].

However, there is a paucity of experimental evidence for the impact of music education programs on reading skills in typically-developing children, perhaps due to the inordinate logistical challenges involved in carrying out longitudinal studies. One correlational study revealed that reading comprehension performance relates with years of music training, even when controlling for additional factors such as age, SES, IQ and number of hours spent reading per week [39], and several longitudinal studies have demonstrated positive effects on reading-related skills using experimenter-designed music training or computer-based training [40]–[46].

Our study is unique, to our knowledge, in assessing reading outcomes in an established music program, using a random-assignment, longitudinal design. Further, our participants were recruited from a low-income, bilingual population, where even modest gains have the potential to make a meaningful difference in academic trajectories. We performed our research in collaboration with Harmony Project, a non-profit organization that has garnered national acclaim for providing free music education to underserved children in Los Angeles for over ten years. This collaboration represented a unique opportunity to assess the impact of an enrichment program that has already proven its viability and sustainability outside the laboratory. Our study participants followed the Harmony Project's standard curriculum, therefore ensuring the ecological validity of our experimental outcomes. Participants were recruited from the Harmony Project waitlist and then randomly assigned to training and control groups, ensuring the groups were equally matched in their motivation to participate in the music program. The lack of comparable alternative enrichment opportunities available to this population made it logistically and economically unfeasible to provide an active control group, however every effort was made to keep control participants and their families engaged with the Harmony community during the course of the study. The training group began music classes immediately after the first assessment and the control group began one year later, after the second assessment. We hypothesized that participation in music class benefits reading ability, predicting that children who received one year of music training would outperform their untrained peers on standardized reading measures.

## Methods

### Ethics Statement

All experimental procedures and forms were approved by the Northwestern University Institutional Review Board.

### Participant information

Participants comprised 42 Spanish-English bilingual elementary school children (26 female), aged 6–9 years (mean age 8.3 years) at first test. All participants had normal hearing at the outset of the study (air conduction thresholds < = 20 dB normal hearing level for octaves from 250–8000 Hz) and this was reassessed after one year to confirm all participants' hearing remained within normal limits. Participants had no known learning, audiological or neurological impairments based on parental report and this was also reassessed after one year. Participants were recruited from the waitlist of Harmony Project and from local elementary schools with active Harmony Project programs, with participation in the research study ensuring a place in a Harmony Project program either immediately following the first assessment or one year into the study. Harmony Project staff maintained regular contact with the families of control participants to promote a sense of engagement with the study and with the Harmony community.

The mission of Harmony Project is to provide free music education to children from low-income communities, and their programs are established in schools where at least 90% of the children qualify for free or reduced lunch. Based on U.S. government guidelines, children qualify for reduced lunch if the family income is 185% or less of Federal poverty guidelines, and free lunch if the family income is less than 130% of poverty level, therefore it can be assumed that the participants in this study were predominantly of low income.

The children were pseudo-randomly assigned to training and control groups following the initial assessment (i.e., the first year of data collection). Minor modifications were made to the assignments to ensure the groups were matched according to age, sex, handedness, IQ, age of English acquisition, English reading ability and maternal education (an index of socioeconomic status: see [47] for discussion regarding the predictive value of maternal education for inferring a child's SES) prior to training (all *p*>0.2 using ANOVA and *X* ^2^ tests, as appropriate, see Table 1).

**Table 1:** Group characteristics before training.

	Training Group	Control Group	Statistic
	(n = 23)	(n = 19)	
Age (years)	mean 7.91 (SD = 0.733)	7.89 (SD = 0.875)	F_(1,40)_ = .005, p = 0.942
Sex	13 females	13 females	χ^2^ = 0.625, p = 0.530
Maternal education (years)	10.14 (4.35)	10.74 (4.07)	F_(1,40)_ = 0.207, p = 0.652
Verbal IQ (T score)	47.13 (10.91)	44.68 (9.050)	F_(1,40)_ = 0.609, p = 0.440
Non-verbal IQ (T score)	53.22 (10.479)	51.21 (12.177)	F_(1,40)_ = 0.329, p = 0.569
Age of acquisition of English (years)	2.04 (1.69)	2.00 (1.73)	F_(1,40)_ = 0.007, p = 0.935

The training group (n = 23) began music classes with Harmony Project after the initial assessment, while the control children (n = 19) remained on the organization's waiting list to begin music classes the following year. None of the participants had previously received music training. Importantly, both training and control groups comprised children wanting to participate in the music program, ensuring that the participants were as well matched as possible for motivation and parental support.

### Testing

Researchers from Northwestern University traveled to Los Angeles to collect data prior to the start of music classes and then again one year later. All testing was carried out in the Harmony Project offices during a three week period. Informed written consent was obtained from caretakers or guardians on behalf of the children participating in the study in either English or Spanish, and informed written assent was obtained in English from the child participants. All forms and experimental procedures were approved by the Northwestern University Institutional Review Board. Participants were monetarily compensated for their testing time.

### Reading proficiency

Reading ability was assessed in English using three standardized literacy measures to capture silent (Test of Silent Word Reading Fluency - TOSWRF) [48] and oral reading speed (Test of Word Reading Efficiency - TOWRE) [49], in addition to phonological processing (Comprehensive Test of Phonological Processing - CTOPP) [50].

The TOSWRF requires the participant to isolate individual words from a contiguous sequence of letters by inserting dividing lines (e.g., dimhowfigblue dim/how/fig/blue). The test is timed (3 minutes) and gradually increases in difficulty.

The TOWRE requires the participant to read aloud lists of real words (Sight subtest) and nonsense words (Phonemic Decoding subtest) as quickly and accurately as possible for 45 seconds. Scores are generated according to the number of correctly named words or nonwords during that time period. A composite reading score was generated by averaging the age-normed TOWRE (“reading efficiency”) and TOSWRF (“reading fluency”) scores.

The CTOPP [50] requires the participant to complete a variety of assessments that establish their ability to perceive and combine sounds into words. The CTOPP is divided into six subtests: Elision (repeat a word while omitting a phoneme located in the beginning or middle of the word – e.g., say cat without/k/), Blending Words (concatenate parts of words into a new whole word), Rapid Number Naming and Rapid Letter Naming (read a list of numbers or letters, respectively, in 30 sec), Nonword Repetition (repeat strings of phonemes that form nonwords), and Number Repetition (repeat strings of numbers). The CTOPP subtests were combined to make three cluster scores: phonological awareness, phonological memory and rapid naming.

### Intelligence

Two-scale IQ was measured through the Wechsler Abbreviated Scale of Intelligence [51], using one verbal and one nonverbal subtest (i.e., Vocabulary and Matrix Reasoning, respectively). The subtest scores are reported separately to differentiate verbal and non-verbal IQ.

### Music Training

The music training consisted of Harmony Project's standard music curriculum: first, students are enrolled in group musicianship classes, meeting for one hour, twice a week. The learning objectives for the musicianship class include basic pitch and rhythm skills, vocal performance, improvisation and composition, and awareness of musical styles and notation as well as basic recorder playing (details provided in Table 2). Based on instrument availability and developmental readiness (assessed by their teacher) students progress to group instrumental classes. The children choose their instruments from currently available options, which depend on teacher and instrument availability. The children in the training group attended Harmony Project programs at one of several different locations within the Los Angeles area and the instrumental class and ensemble opportunities varied across locations (details are provided in Table 3), with children typically participating in 4–5 hours of instrumental classes per week. The children in the control group went about their normal routines and did not engage in musical activities over the course of the year; this was confirmed when the participants returned for testing the following year.

**Table 2:** Musicianship Class: Learning Objectives.

**Rhythm**
Identify, read and perform basic note/rest values, measures, bar lines, meters (e.g. music math)
Identify and perform simple rhythmic patterns and ostinatos with a steady beat
**Pitch**
Name lines/spaces on the staff (treble and bass clef, basic understating of grand staff and C clef)
Identify and perform simple melodic patterns
Match and adjust pitch
Follow pitch direction through movement
Sing and identify major and minor scales
**Performance**
Sing and perform independently and in groups, on pitch and in rhythm, blending timbres
Follow a conductor for dynamics, tempo, and cues
Exhibit appropriate rehearsal etiquette
Students echo short rhythms and melodic patterns (call and response)
**Improvisation and Composition**
Improvise "answers" in the same style to given rhythmic and melodic phrases (another form of call and response)
Improvise simple rhythmic variations and melodic embellishments on familiar melodies
Create and arrange short songs and instrumental pieces within specified guidelines
Write and perform simple compositions
**Musical Awareness**
Explain personal preferences for music and styles using appropriate terminology for music, music notation, music instruments and voices
Identify instruments and their sounds, including instruments from various cultures
Listen to music, analyze and describe structure/emotion
**Musical Terms**
Melody, rhythm, and harmony
Beat, measure, bar line, repeat sign, double bar
Tempo (lento, adagio, allegro & presto)
Dynamics (p, mp, mf, f, crescendo and decrescendo)
Whole, half, dotted half, quarter, and eighth notes;
Whole, half, quarter rests
Time signature (4/4, 2/4, 3/4)
Verse, chorus
Tutti, solo, duet
Scale
Chord
Grand Staff, treble clef, bass clef, C clef
Sharp, flat
Key signature
Intonation
**Orchestra Instrumentation**
Conductor
Strings: Violin, Viola, Cello, Double Bass, Guitar, Harp
Winds: Recorder, Flute, Clarinet, Oboe, Bassoon, Saxophone
Brass: French horn, Trumpet, Trombone, Tuba
Percussion: Timpani, Cymbals, Snare Drum, Drum Set, etc.
Piano and Keyboard

**Table 3:** Summary of instrumental programs and instruments played.

Harmony Project program	Typical weekly class participation	Number of children
Alexandria Elementary School	One-hour instrumental classes twice a week plus a two hour string ensemble rehearsal each week	3
Beyond the Bell	Twice-weekly two-hour ensemble rehearsals. These include pull-out sectional rehearsals, which are similar to large instrumental classes at other sites.	9
EXPO Center (YOLA)	One-hour instrumental music classes each week and a three hour ensemble rehearsal each week.	3
Hollywood	One-hour instrumental classes twice a week plus a three-hour ensemble rehearsal (concert band) each week.	4
		**19**
**Instruments played**		
*These students graduated to instrumental instruction in January 2012:*		
Bass		3
Cello		2
Trumpet		8
Viola		1
*These students continued musicianship class until the end of the academic year and began instrumental instruction in fall 2012:*		
Musicianship/recorder		5
		**19**

### Data analyses

Initial group comparisons were made at the outset of the study using independent samples t-tests, to confirm no pre-existing differences between the groups (Table 1). The effect of music training on reading performance was assessed using a repeated measures ANOVA with music training group as the fixed factor and year of study (i.e. pre- and post-training for the training group) as the within-subject factor. Post hoc, we performed paired samples t-tests to determine the change in reading measures over time within each group separately, as well as Pearson's correlations to assess relationships between change in performance on the composite reading score and CTOPP cluster scores across participants. Normality for all data was confirmed by the Shapiro–Wilk test for equality. All statistical analyses were performed using SPSS (SPSS Inc., Chicago, IL) and reflect two-tailed values.

## Results

**Figure 1:**
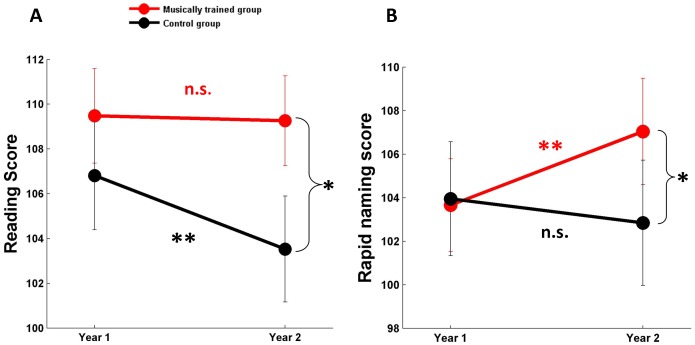
Music training supports reading abilities and rapid naming. (A) The children who received music training (n = 23) maintained their age-normed level of reading performance after one year (year 1: 109.5+/−2.1 (mean +/−1 S.E.), year 2: 109.3+/−2.0), while the untrained children's scores declined (n = 19) (year 1: 106.8+/−2.4, year 2: 103.5+/−2.4). (B) The musically-trained group improved on rapid naming (year 1: 103.7+/−2.1, year 2: 107.0+/−2.4), while the untrained controls showed no improvement (year 1: 103.9+/−2.6; year 2: 102.8+/−2.9). The groups did not differ on either measure at the outset of the study.

The groups did not differ at the outset of the study on CTOPP, TOWRE or TOSWRF measures (p>0.2 for paired t comparisons of each individual subtest, as well as the composite reading score). We found a modest but significant effect of music training on year-over-year reading performance (RMANOVA group x year interaction: F_(1,40)_ = 5.358, p = 0.026, partial eta-squared  = 0.118; Figure 1A). Post-hoc analyses showed that the children who received music training maintained their age-normed performance on the composite reading measure after one year (paired *t*_(22)_ = 0.251, p = 0.804; year 1: 109.5+/−2.1 (mean +/−1 S.E.), year 2: 109.3+/−2.0), while the untrained group's scores declined significantly (paired *t*_(18)_ = 3.237, p = 0.005; year 1: 106.8+/−2.4, year 2: 103.5+/−2.4).

**Figure 2:**
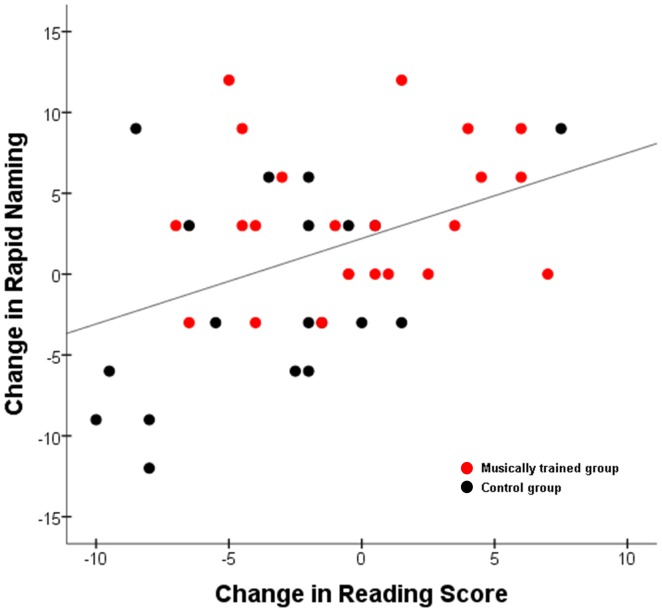
Improvement in rapid naming relates to reading improvement. Year-over-year improvement in rapid naming was correlated with the change in composite reading score across all participants (r = .412, p = .007, n = 42).

There was also a significant effect of music training on rapid naming performance (RMANOVA group x year interaction: F_(1,40)_ = 7.246, p = 0.010, partial eta squared  = 0.153; Figure 1B). In this case, the musically-trained group improved on rapid naming (paired *t*_(22)_ = −3.574, p = 0.002; year 1: 103.7+/−2.1, year 2: 107.0+/−2.4) while the untrained controls showed no improvement (paired *t*_(18)_ = 0.769, p = 0.452; year 1: 103.9+/−2.6; year 2: 102.8+/−2.9). The year-over-year improvement in rapid naming correlated with the change in composite reading score across the group (r = 0.412, p = 0.007; Figure 2). Phonological awareness and phonological memory performance did not change significantly in either group (phonological awareness: RMANOVA main effect F_(1,40)_ = 0.158, p = 0.693; group x year interaction: F_(1,40)_ = 0.602, p = 0.442; phonological memory: RMANOVA main effect F_(1,40)_ = 0.233, p = 0.632; group x year interaction F_(1,40)_ = 0.643, p = 0.428). The raw data for these analyses can be found in Table S1.

## Discussion

We show that musically-trained children maintained their age-normed level of reading ability after one year whereas a matched control group's performance deteriorated over the same time period. While we did not see a *positive* improvement in reading ability in the trained group, we interpret the decline in age-normed reading scores in our passive control group as consistent with the expected negative trajectory of performance in a low-income population [52]. We interpret these modest outcomes as evidence that the auditory enrichment provided by participation in music may help to keep literacy development on track, counteracting the negative impact of low socioeconomic status.

There are several mechanisms by which engagement in music could influence reading ability. While reading is not primarily an auditory activity, the development of reading skills depends heavily on auditory perception and the ability to parse out meaningful speech elements from an auditory stream. Reading also relies on general cognitive functions such as working memory, and upon the ability to map visual symbols to sounds, which has previously shown improvement with computerized music-based training programs [46]. In the present study we observed training effects specifically in the measures of reading fluency and rapid naming, but neither in phonological memory nor phonological awareness. It is possible that playing music promotes reading fluency by giving children additional practice mapping visual symbols to the production of sounds, as they learn to play their instrument from a written score. This “extra reading practice” in the context of reading musical notation may help to strengthen some of the same cognitive and integrative processes involved in word reading [53].

The lack of a training effect on phonological awareness in the present study is consistent with the findings of Moreno et al (2011), although a smaller-scale study did show strengthened phonological awareness with music training in preschoolers [54]; it is possible that the younger age of the participants may have resulted in a greater impact of training in this smaller study. There is also evidence that early bilingual experience may confer an advantage for phonological awareness in English-Spanish bilinguals compared with monolinguals [55], and it is therefore possible that the additional auditory enrichment provided by music training had less of an effect on phonological awareness in our population due to the preexisting influence of bilingual experience.

Prior research has been inconclusive in terms of the extent to which rapid naming, a measure of processing speed, can be improved with training [56] and, to our knowledge, this study is the first to demonstrate that rapid naming performance can be affected by *music* training in particular. Rapid naming performance during early reading development has been identified as a unique and significant predictor of later reading ability [57], [58]. It therefore seems that musical activities may support reading proficiency not only by providing additional reading practice [53], but also by strengthening core abilities such as processing speed.

Another mechanism by which music training may influence reading development is by strengthening temporal sequence processing. The natural rhythms of speech provide a temporal framework that guides attention and facilitates processing of both semantic and syntactic structure in the speech signal [59]–[62]. Rhythm-related skills have been associated with reading and pre-reading abilities in both typically-developing and impaired children [16], [34], [35], [63]–[71], and rhythm-based training programs have demonstrated some success in improving reading skills [17]–[19], [72]–[74]. Rhythm skills have also been shown to be strengthened in musically-trained children [75], especially in those who are trained early in life (before the age of 7) [76].

There is evidence that rhythm and reading abilities call upon common neural resources [16], [70], [71], [77], and that both depend upon the temporal precision and consistency of neural responses to sound [71], [77]–[79]. While our data cannot address these issues directly, previous research demonstrates that musicians have more precise timing and more consistent neural responses than non-musicians [38], [80]–[82], and it has been proposed that music training may strengthen reading ability by increasing underlying neural consistency and thereby supporting critical reading-related sub-skills, including rhythm perception, phonological awareness and auditory working memory (see [16], for review). Recent research has revealed that adolescents from low socioeconomic backgrounds have less consistent neural responses to speech than their high-SES peers [83]; it is therefore an important direction for future research to determine whether the auditory enrichment provided by music training could help to offset negative consequences of impoverishment on underlying neural function.

The development of pitch-related skills is another important component of music training that may influence reading skills. A 2002 study demonstrated that in five year-olds, pitch but not rhythm skills were predictive of beginning reading skills and related with phonemic awareness [84], replicating previous findings from a smaller study [85]. Research with adults suggests that the perception of complex pitch patterns and melodic contour may be especially important to the development of reading skills because it aids in the detection of stress patterns and facilitates the segmentation of a continuous speech stream into individual phonemes [86].

It was not logistically feasible in this study to include an *active* control group, due to the lack of comparable alternative enrichment opportunities in these communities, and the control children went about their usual routine without participation in any additional activities. It is therefore impossible to conclude from these data that musical engagement is the only or best enrichment strategy since other programs could provide similar benefits. However, both music and language involve meaningful communication through sound, and there is evidence for specific links between music and literacy skills [41], [84], [87]–[91], including research comparing different forms of arts-based education programs (e.g. music and painting classes), in which improvements in language skills were found following participation in music training but not for other forms of arts enrichment [28], [30], [35]. Taken together, these outcomes suggest that music training may confer benefits for languages skills above and beyond other arts-based activities.

Reading interventions directly targeting specific sub-skills, such as phonological awareness, have been effective in improving the trajectory of reading development in low-income populations [92], however a challenge with any focused intervention is ensuring the effects are long-lasting. The development of a child's intrinsic motivation to read is an important factor in ensuring the long-term efficacy of reading interventions [93], [94], and previous research indicates that a student's reading motivation is an even stronger predictor of reading outcomes than family background [95], suggesting that programs designed to increase reading motivation could be particularly effective in reducing the achievement gap between children from low and high-SES backgrounds.

It is possible that participation in an engaging music program may influence the development of reading skills by increasing a child's overall motivation to learn, and that observed benefits are therefore not the result of playing music, per se. However, increasing student motivation is not a trivial accomplishment: one of the unique characteristics of music is its ability to engage an individual on many levels, socially, emotionally, intellectually and creatively, promoting other aspects of development such as self-confidence and discipline [96] and fostering social cohesion [97]. These are positive effects in themselves, particularly in at-risk communities, and they may also provide the key to why music is such a powerful teacher. Music engages the emotional circuitry of the brain [98], [99], which promotes neural plasticity [100], [101]. Importantly, music is a form of enrichment that can be sustained over a lifetime: children who participate in musical activities are more likely to participate as adults [3]; even if they do not continue playing music, the positive biological impact of early music training may be preserved into adulthood, many years after training has ceased [102], [103].

Children from low-income communities face many obstacles to literacy development that music programs cannot directly address; however, by engaging auditory, cognitive and communicative skills that are also important for reading, music may help to offset some of the negative consequences of low socioeconomic status. The paucity of longitudinal work in this area means that little is known about how musical experience influences reading-related skills over the course of development. Further, it has been suggested that the current understanding of how auditory perception relates to reading skills may have been impeded by the failure to account for musical experience [104]. In other words, understanding the effects of music training is important not only in assessing the educational benefits of music programs, but in gaining a clearer understanding of the mechanisms that underlie reading, since the relations between reading ability and related skills may themselves be altered by musical experience.

Both music and reading are complex activities, and there is much still to be learned about which aspects of language processing might be shaped by musical experience, and which may not [105]. Further research is needed to elucidate the relations between reading skills and distinct aspects of musical experience (e.g. rhythm vs. pitch), and how these interact with other factors influencing reading development such as home literacy environment, language exposure and bilingual experience. As more research is done to understand common mechanisms underlying music and literacy skills, this knowledge can help to inform the development of music programs to provide maximum benefit for individuals and communities in terms of personal, social and academic as well as artistic development.

## Conclusions

In summary, our study demonstrates a significant effect of group music training on reading development over the course of one year: children who received one year of music instruction maintained their age-normed reading levels, whereas a matched control group showed a modest decline in performance. Music programs have a great deal to offer in their own right, and our findings are consistent with the interpretation that engagement in music programs such as Harmony Project may also help to maintain reading performance in low-income populations where declines are typically expected.

Scientifically rigorous assessments of existing music programs present significant logistical challenges, but laboratory-based research must continue to work in tandem with “real world” studies to develop a full understanding of how music education can support literacy skills. We present these modest outcomes as an important contribution to the literature and encourage further research both to quantify the negative impact of poverty on reading development, and to assess the potential for arts-based enrichment programs to counteract those declines.

## Supporting Information

Table S1 **Raw data for the analyses reported in this article.** Data collected in the second year of the study are labeled with the prefix “B”.
(XLS)
